# Adverse treatment outcomes in multidrug resistant tuberculosis go beyond the microbe-drug interaction: Results of a multiple correspondence analysis.

**DOI:** 10.7705/biomedica.5072

**Published:** 2020-12-09

**Authors:** Ángela Tobón, Johana Rueda, Diego H. Cáceres, Gloria I. Mejía, Elsa M. Zapata, Fernando Montes, Antonio Ospina, Santiago Fadul, Lizeth Paniagua, Jaime Robledo

**Affiliations:** 1 Unidades de Bacteriología y Micobacterias y Micología Médica y Experimental, Corporación para Investigaciones Biológicas, Medellín, Colombia Corporación para Investiga. Biológicas Corporación para Investigaciones Biológicas Medellín Colombia; 2 Programa de Control de Tuberculosis, Secretaría de Salud del Municipio de Medellín, Medellín, Colombia Secretaría de Salud del Municipio de Medellín Medellín Colombia; 3 Grupo de Transmisibles - Equipo de Respiratorias, División de Vigilancia y Análisis del Riesgo en Salud Pública, Instituto Nacional de Salud, Bogotá, D.C., Colombia Instituto Nacional de Salud BogotáD.C Colombia; 4 Programa de Control de Tuberculosis, Secretaría de Salud y Protección Social de Antioquia, Medellín, Colombia Secretaría de Salud y Protección Social de Antioquia Medellín Colombia; 5 Escuela de Ciencias de la Salud, Universidad Pontificia Bolivariana, Medellín, Colombia Universidad Pontificia Bolivariana Universidad Pontificia Bolivariana Medellín Colombia

**Keywords:** Tuberculosis, multidrug-resistant, extensively drug-resistant tuberculosis, treatment outcome, tuberculosis fármacorresistente, falla del tratamiento, tuberculosis resistente a medicamentos, tuberculosis extensamente resistente a fármacos

## Abstract

**Introduction:**

Multidrug-resistant tuberculosis treatment is effective in 50% of patients due to several factors including antibiotic susceptibility of the microorganism, adverse treatment reactions, social factors, and associated comorbidities.

**Objectives:**

In this study, we describe the demographics, clinical characteristics, and factors associated with treatment outcomes in multidrug-resistant tuberculosis (MDR-TB) patients in Medellín, Colombia.

**Materials and methods:**

We conducted a retrospective analysis using data from patients diagnosed with MDR-TB attending *Hospital La María* in Medellín, Colombia, for treatment between 2010 and 2015. Patients were categorized as having successful (cured) or poor (failure, lost to follow-up, and death) treatment outcomes. Associations between demographic, clinical factors, laboratory results, treatment outcomes, and follow-up information were evaluated by univariate, multivariate, and multiple correspondence analyses.

**Results:**

Of the 128 patients with MDR-TB, 77 (60%) had successful outcomes. Of those with poor outcomes, 26 were lost to follow-up, 15 died, and 10 were treatment failures. Irregular treatment, the presence of comorbidities, and positive cultures after more than two months of treatment were associated with poor outcomes compared to successful ones (p<0.05 for all). The multiple correspondence analyses grouped patients who were lost to follow-up, had HIV, and drug addiction, as well as patients with treatment failure, irregular treatment, and chronic obstructive pulmonary disease.

**Conclusion:**

The recognition of factors affecting treatment is essential and was associated with treatment outcomes in this series of patients. Early identification of these factors should increase the rates of treatment success and contribute to MDR-TB control.

## Resumen

**Introducción.:**

El tratamiento de la tuberculosis multirresistente tiene una efectividad del 50 %, afectado por múltiples factores como la sensibilidad del microorganismo, las reacciones secundarias, los factores sociales y las comorbilidades existentes.

**Objetivos.:**

Describir la demografía, las características clínicas y los factores pronósticos asociados con los resultados del tratamiento en pacientes multirresistentes (TB-MDR) de Medellín, Colombia.

**Métodos.:**

Se hizo un análisis retrospectivo de los datos de los pacientes con TB-MDR atendidos en el Hospital La María de Medellín, Colombia, que fueron tratados entre el 2010 y el 2015. Los pacientes se categorizaron con tratamiento exitoso (curados) o con tratamiento fallido (falla en el tratamiento, pérdida durante el seguimiento y muerte). Se determinó la asociación entre las características demográficas y clínicas, los resultados de los exámenes de laboratorio, los desenlaces del tratamiento y la información del seguimiento, utilizando análisis univariado, multivariado y de correspondencia múltiple.

**Resultados.:**

De 128 pacientes con TB-MDR, 77 (60 %) tuvieron un tratamiento exitoso. De los que tuvieron un tratamiento fallido, 26 pacientes se perdieron en el seguimiento, 15 murieron y 10 tuvieron falla en el tratamiento. El tratamiento irregular, las comorbilidades y los cultivos positivos más allá de 2 meses de tratamiento se asociaron significativamente con los tratamientos fallidos (p<0,05). El análisis de correspondencia múltiple agrupó los pacientes con pérdida en el seguimiento, con HIV y tratamientos irregulares, y los pacientes con tratamientos irregulares y enfermedad pulmonar obstructiva crónica con falla en el tratamiento y muerte.

**Conclusión.:**

El reconocimiento temprano de los factores que afectan el desenlace del tratamiento de los pacientes con TB-MDR es esencial; la identificación de dichos factores debería incrementar el éxito del tratamiento y contribuir al adecuado control de la TB-MDR.

Multidrug-resistant tuberculosis (MDR-TB) continues to be an obstacle for disease control. It has been estimated that globally, 4.1% of new tuberculosis cases and 19% of previously treated cases have MDR-TB. In other words, there are 490,000 new MDR-TB cases worldwide annually. Additionally, 6.2% of MDR-TB cases are extensively drug-resistant (XDR) ^1^^,^^2^.

Colombia, with a population of 45.5 million, reported 13,870 tuberculosis cases in 2017; 420 cases were reported as drug-resistant and 108 as MDR-TB, out of which 2.4% were new cases and 14% had been previously treated ^2^. According to the national tuberculosis program, 32 XDR-TB cases have been diagnosed in the country to date ^3^. The department of Antioquia reported a fifth of the national MDR-TB cases, half of them in Medellín, the capital city ^4^.

MDR-TB cases pose problems for tuberculosis control programs: Increase in treatment costs, longer duration of treatment, and less efficacy. Likewise, this treatment is associated with more adverse effects than that required for a regular tuberculosis patient. Adverse effects, in turn, are accompanied by drug availability constraints, lack of treatment compliance, and administrative barriers for delivering, as well as for treatment follow-up ^5^. In this context, the global treatment cure rate for MDR-TB is 54%, mostly due to mortality and lack of follow up. Only 30% of XDR-TB patients in the 2014 cohort reported from 138 countries completed their treatment successfully ^1^. From 2000 onwards, the World Health Organization (WHO) and the Stop TB Partnership provided support to countries for MDR-TB management through the Green Light Committee. This initiative was created to evaluate, lend guidance, and facilitate access for tuberculosis control programs to second-line drugs at a reduced price with an assured quality ^5^.

In Colombia, an MDR-TB treatment program was initiated in 2010 in Medellín to improve the management of these patients with second-line antituberculosis drugs. It was supported by the Green Light Committee and the national tuberculosis program [a public-private alliance formed by the *Dirección Seccional de Salud, Secretaría de Salud de Medellín, Hospital La María, Corporación para Investigaciones Biológicas, Liga Antituberculosa de Antioquia,* and *Fundación Red de Apoyo Social* (RASA)].

In this study, we describe the demographics, clinical characteristics, and prognostic factors associated with treatment outcomes in these MDR-TB patients. Given the lack of published studies on the treatment results of these patients in the country, we described a cohort of patients treated for MDR-TB and analyzed the socio-demographic factors associated with their outcomes.

## Materials and methods

We conducted a descriptive retrospective study based on the data regarding the diagnosis, treatment, and outcomes of MDR-TB patients enrolled in a national program for improving MDR-TB treatment and management. It included 146 patients diagnosed with MDR-TB at *Hospital La María* in Medellín who started and received treatment from November, 2010, to June, 2015. We included the data from 128 patients in the analysis and we defined their final treatment outcome as follows: Patients with a successful treatment were those declared cured according to microbiological and clinical parameters while patients with a poor treatment outcome included those whose treatment failed, those who died (associated with their tuberculosis condition), and those lost to follow-up ^6^.

Patients included in the program were previously diagnosed with MDR-TB using a GenoType MTBDRplus™ *(Hain* Life Science GmbH, Nehren, Germany) molecular test at the Regional Public Health Laboratory. After diagnosis and before starting treatment, a new sputum sample was processed using a decontamination procedure standard method ^7^. From the digested sputum a direct smear was stained with auramine-rhodamine and 0.1 ml were inoculated in Lówenstein-Jensen medium (L-J) and 0.5 ml in a mycobacterial growth indicator tube (MGIT) (Becton, Dickinson, and Company, Sparks, MD, USA). In cultures identified as positive, a smear stained by Kinyoun was carried out and subcultured on thin layer agar. Final identification of *Mycobacterium tuberculosis* was based on the inhibition of *M. tuberculosis* complex growth by p-nitrobenzoic acid in thin layer agar and a standard phenotypic test; resistance to isoniazid and rifampin was also confirmed using thin layer agar ^8^.

Seventy-nine MDR-TB isolates were tested for susceptibility to second-line drugs using the BD BACTEC MGIT 960™ system ^9^. Drug stock concentrations were prepared following standard recommendations ^10^^,^^11^. Patients were given the standard treatment ^12^ with kanamycin, levofloxacin, cycloserine, pyrazinamide, and ethionamide for 18 to 24 months. Clinical and laboratory follow-up was done monthly, as well as an audiometry test at three months and at the end of the administration of the injectable medication while thorax PA and lateral X-rays were done every six months. Changes in the treatment were introduced due to drug intolerance, drug resistance, or the presence of serious adverse effects. The alternative drugs used were *p*-aminosalicylic acid, ethambutol, moxifloxacin, amoxicillin-clavulanate, and linezolid.

The demographic characteristics, clinical results, laboratory results, treatment outcomes, and follow-up information were collected from each patient's clinical record using a data collection form. All data were entered into a Microsoft Excel database.

To identify differences in means, Student's t test or Mann-Whitney U test was used. Odds Ratio (OR) and their 95% confidence intervals (CI) were calculated. A p-value of less than 0.05 defined significant differences. Variables with a p<0.05 were used in a logistic regression model to adjust independent factors associated with treatment outcome.

We performed a multiple correspondence analysis to evaluate the relationships among treatment outcomes and patients' baseline conditions: human immunodeficiency virus (HIV) infection, drug addiction, irregular treatment (as evidenced by failing to finish treatment or intermittent treatment without completion of the full treatment scheme), chronic obstructive pulmonary disease (COPD), diabetes mellitus, and undernourishment. The analysis considered the level of significance (weight) of each factor to explain the total sample variability (inertia). All analyses were performed using Epidat 3.1 and STATA 11.0™.

The study was conducted in accordance with the national public health regulations as part of a pilot program for MDR-TB treatment supported by the Green Light Committee and the national tuberculosis program. As such, no additional ethical approval was necessary. Individual participants gave written informed consent before starting treatment according to the country regulations and the institutional consent was obtained for records review. All patient information was anonymized before analysis.

## Results

We analyzed the data from 128 patients treated for MDR-TB reporting treatment outcomes. The patients had a mean age of 40 years and 69% (n=88) of them were male.

Out of the 128 patients, 51 (40%) had an irregular treatment (41 with poor adherence, seven due to problems in drug supply, and three for both reasons), and 112 (88%) had risk factors for MDR before being treated for MDR-TB. Out of these patients, 49 (44%) had previously received irregular anti-tuberculosis treatment, 46 (41%) had previously received regular anti-tuberculosis treatment, and 17 (15%) had had contact with a tuberculosis patient. A variety of adverse effects were found in these patients, mostly diarrhea (three patients), vomit (four patients), hypoacusis (13 patients), and gastritis (15 patients).

Thoracic surgery was performed in eight patients (6%), six of them with a successful outcome. The type of surgery practiced was upper lobectomy in four patients, upper bilobectomy in one, and pneumonectomy in three.

Forty-eight percent of patients had an associated comorbidity: drug addiction (n=18; 14%), diabetes mellitus (n=16; 13%), HIV co-infection (n=14; 11%), COPD or undernourishment (n=10; 8% each), and other comorbidities (n=16; 13%).

**Figure 1:**
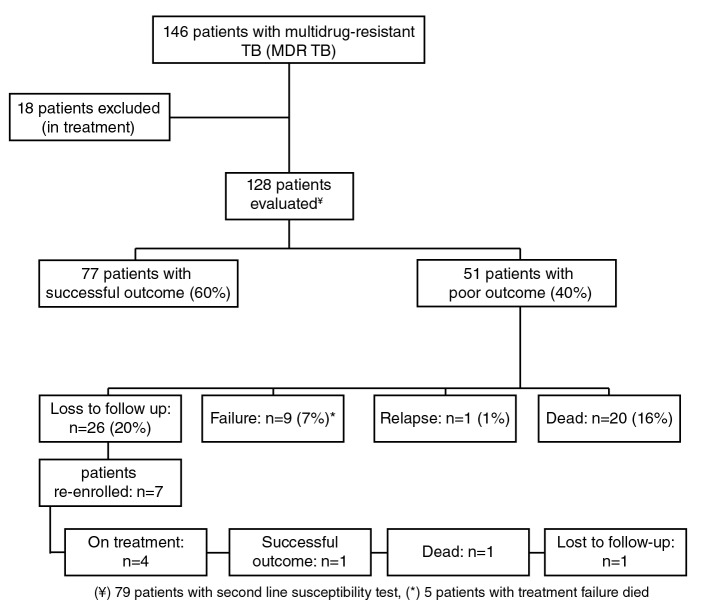
Flow chart of analyzed patients with MDR-TB

A total of 77 (60%) MDR-TB patients had successful treatment outcomes, 26 (20%) were lost to follow-up, 15 (12%) died, and 10 (8%) had a failure during treatment (table 1 and figure 1).

**Table 1:** Demographics, clinical characteristics, and treatment outcomes of 128 patients with multidrug-resistant tuberculosis

Characteristics	Patients n (%)
Total	128
Male	88 (69)
Age, years, median (range)	40 (16-80)
Time of treatment in months, median (range)	18 (1-28)
Time of positive culture in months, median (range)	1 (1-10)
Irregular treatment	
Yes	51 (40)
No	77 (60)
Risk factors for MDR-TB	
Yes^†^	112 (88)
No	16 (12)
Thoracic surgery	
Yes	8(6)
No	120 (94)
Co-morbidities	
HIV	14(11)
Diabetes	16 (13)
Drug addiction	18 (14)
COPD	10(8)
Undernourishment	10(8)
Other	16 (13)
Patient outcome	
Successful outcome	77 (60)
Lost to follow up	26 (20)
Failure ^*^	10(8)
Death	15(12)

MDR-TB: Multidrug-resistant tuberculosis; HIV: Human immunodeficiency virus; COPD: Chronic obstructive pulmonary disease

* Five patients died while receiving treatment.

(¥) 79 patients with second line susceptibility test, (*) 5 patients with treatment failure died

In the univariate analysis, an irregular treatment and the presence of comorbidities had a significant association with poor outcomes (OR=5.14; p<0.001 and 4.05; p<0.001, respectively), as well as drug addiction and HIV infection (OR=4.92; p=0.005 and 4.45; p=0.017, respectively). Patients with poor outcomes took more than two months to achieve microbiological sterilization (OR=1.26; p<0.026) (table 2). Sixty-five patients (85%) with a successful outcome had negative cultures after two months while only 30 patients (60%) with poor outcomes had them (p=0.0015).

**Table 2:** Characteristics of 128 patients with multidrug-resistant tuberculosis and their association with treatment outcomes

Variable	Poor outcome (n=51)	Successful outcome (n=77)	Univariate		Multivariate	
			OR (CI 95%)	p	OR (CI 95%)	p
Age (years)^*^	41 (15)	40 (14)	1.00 (0.98-1.02)	0.737	-	
Sex (male)^†^	33 (65)	55 (71)	0.73 (0.34-1.56)	0.422	-	
Irregular treatment^†^	32 (63)	19 (25)	5.14 (2.38-11.1)	>0.001*	9.38 (2.30-38.2)	0.002*
Time of treatment (months)^*^	5 (12)	18 (2)	0.79 (0.73-0.85)	>0.001*	0.66 (0.57-0.77)	>0.001*
Risk factor of MDR^†^	46 (90)	66 (86)	1.53 (0.49-4.70)	0.455	-	
Co-morbidities^†^	35 (69)	27 (35)	4.05 (1.90-8.61)	>0.001*	2.33 (0.88-5.65)	0.089
HIV^†^	10 (20)	4 (5)	4.45 (1.31-15.1)	0.017*	3.26 (0.77-13.78)	0.108
Diabetes^†^	7 (14)	9 (12)	1.20 (0.41-3.46)	0.733	-	
Drug addiction^†^	13 (25)	5 (6)	4.92 (1.63-14.8)	0.005*	1.56 (0.42-5.73)	0.089
COPD^†^	2 (4)	8 (10)	0.35 (0.07-1.73)	0.199	-	
Undernourishment^†^	6 (12)	4 (5)	2.43 (0.65-9.09)	0.186	-	
Thoracic surgery^†^	2 (4)	6 (8)	0.48 (0.09-2.49)	0.385	-	
Time of positive culture (months)^*^	2 (3)	1 (1)	1.26 (1.02-1.56)	0.026*	1.85 (1.29-2.64)	0.001*

OR: Odds Ratio: (CI) 95% confidence interval

* Median (interquartile range)

† Number (%)

** Statistically significant differences (p<0.05).

The subgroup of patients with diabetes mellitus needed more than two months for negative culture conversion compared to those without diabetes mellitus (p=0.028). Nine patients with diabetes mellitus had a successful outcome and seven had poor outcomes; four of these seven patients died, two had treatment failure, and one was lost to follow-up.

Using a forward selection stepwise logistic regression model, we identified two factors associated with poor outcomes: Previous irregular treatment (OR=9.38, 95% CI: 2.30-38.2, p=0.002) and time (months) of positive culture after treatment initiation (OR=1.85, 95% CI: 1.29-2,64, p=0.001). The duration of tuberculosis treatment for 18 months was associated with a successful outcome (OR=0.79, 95% CI: 0.73-0.85, p=0.001) (table 2).

Out of 128 patients, 79 (62%) had a susceptibility test for second-line drugs. Of these, nine (11%) showed resistance exclusively to isoniazid and rifampicin, 70 (89%) had additional resistance to one or more second-line drugs, 54 (68%) were resistant to ethionamide, 49 (62%) to pyrazinamide, and 12 (15%) to both drugs. We found a pre-XDR-TB condition in 12 (15%) patients, and five patients had developed XDR-TB; four of them died, including one diabetic patient who had XDR-TB from his admission. All XDR-TB patients had a positive culture at the time of death or loss to follow-up. Seventy-seven patients completed successful treatment, 68 of them received cycloserine, 60 levofloxacin, and 53 kanamycin. Due to the resistance to ethionamide or pyrazinamide, or to cycloserine adverse effects, scheme changes were necessary: 13 patients received linezolid, 41 amoxicillin/ clavulanate, and 2 XDR-TB patients received imipenem.

**Figure 2:**
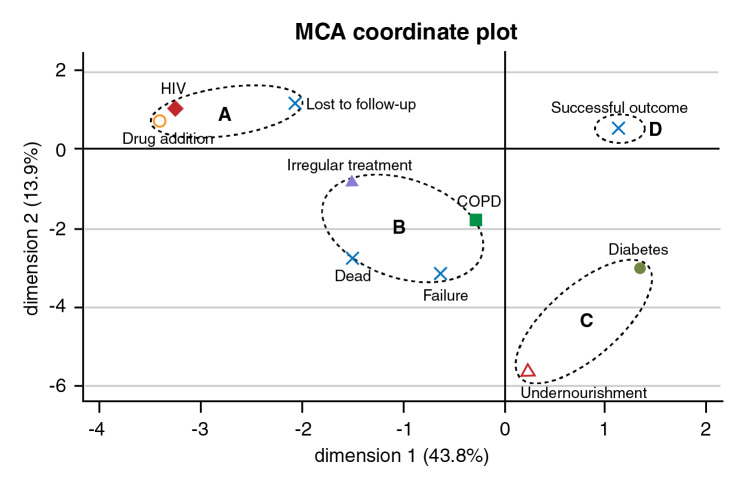
Multiple correspondence analyses between treatment outcomes and patient's base line conditions. Based on the patient's baseline conditions, the multiple correspondence analysis showed several patterns: (a) Lost of follow-up was associated with HIV infection and drug addiction, (b) Death and treatment failure were associated with patients with irregular treatment and COPD. (c) Diabetes and undernourishment were not directly associated with any of the outcome categories defined in this study, (d) A successful outcome was not associated with any of the patients' baseline conditions described in this study. The horizontal and vertical axes represent the first and second principal components.

Four groups were identified using the MCA analysis (figure 2). The first group (a) comprised those patients lost to follow up whose associated baseline conditions were HIV infection and drug addiction. The second group (b) included patients who died or presented a treatment failure and showed an association with irregular previous treatment and COPD. The third group (c) comprised patients with diabetes and undernourishment and were not directly associated with any of the outcome categories defined in this study. Finally, the fourth group (d) included patients with a successful outcome and was not associated with any of the baseline conditions analyzed in this study (figure 2).

## Discussion

In this study, 60% of 128 MDR-TB patients had a successful outcome. Overall, treatment outcomes for this population were consistent with previously reported outcomes. In a systematic review of 26 trials with a total of 4,959 MDR-TB patients, 62% of them met the definition of successful treatment ^13^. This 60% treatment success rate for our population was higher than the rate of 54% reported worldwide by WHO ^1^ but less than the 86% achieved by individualized MDR-TB treatment in the study by Bolhuis, *et al.*^14^.

In our study, 26 (20%) patients were lost to follow-up and seven of these re-initiated their treatment until completion. WHO has reported a global rate for lost to follow-up of 15% ^1^. The multiple correspondence analyses analysis in the present study showed that the loss to follow-up is a poor treatment outcome associated with HIV infection and drug addiction. One study in Africa reported that 56% of HIV patients were lost to follow-up during MDR-TB treatment suggesting that better patient-tracking activities, an improved understanding of the reasons that generate loss to follow-up outcomes, and an earlier initiation of antiretroviral therapy are needed to improve treatment completion ^15^. In Georgia, drug addiction was reported to be significantly associated with a higher risk of loss to follow-up ^16^. In our study, the non-inclusion of patients lost to follow-up in the analysis would increase the success of the treatment rate from 60% (77/128) to 75% (77/102). Multiple correspondence analyses analysis also identified poor treatment outcomes associated with COPD and previous irregular treatment. Several studies have associated COPD ^17^ and previous treatment as independent predictors of death in MDR-TB patients ^18^ or poor treatment outcomes ^19^.

Surgery has been recommended for the treatment of patients with MDR-TB who do not respond to drug treatment and have a persistent cavitary well-localized lesion but adequate respiratory function, as well as when not enough active drugs are available for a curative scheme ^20^. In our study, eight patients underwent lung resection surgery with successful outcomes in six of them. A systematic review and meta-analysis with data from 24 studies revealed a significant association between surgical intervention and successful treatment compared to non-surgical interventions (OR=2.24, 95%CI 1.68-2.97) ^21^.

Sputum smears and cultures are direct indicators of bacteriological load, the patient's infectious status, and, ultimately, the success of treatment. Conversion of initial sputum culture in the first three months is essential to label the patient as non-infectious, which, in turn, is an important indicator to determine whether or not it is necessary to extend the time of treatment ^22^. In this study, most patients with a successful outcome (85%) had a sputum culture negative conversion at two months while 60% of those with a poorer outcome had negative cultures in the same period of time. This is consistent with studies in which sputum culture conversion within two months was considered as a marker of a successful outcome in HIV negative patients ^23^.

In this paper, patients with diabetes mellitus needed more than two months for negative culture conversion compared with those who did not have diabetes mellitus (p=0,028). This finding suggests that besides optimal control of diabetes mellitus, it would be necessary to extend the recommended duration of treatment in order to avoid treatment failure, relapses, and deaths. This situation should be addressed when considering the use of a new standardized shorter treatment regimen for patients with MDR-TB ^24^.

Despite effective anti-tuberculosis chemotherapy, case-fatality rates of up to 25% have been described in patients with MDR-TB in both industrialized and resource-poor settings ^25^. In the present study, 16% of MDR-TB patients died, four of whom had XDR-TB. The cause of the increased death risk in patients with MDR-TB is diverse but commonly associated with HIV infection, diabetes mellitus, and lifestyles such as alcohol abuse, smoking, and illicit drug use ^25^. Among the 20 patients in our study who died while on anti-tuberculosis treatment, 17 had comorbidities, mainly HIV, diabetes mellitus, and illicit drug use.

A common characteristic of isolates from patients was the increased resistance to pyrazinamide (62%) and ethionamide (68%). A systematic review and meta-analysis found that 61% of patients with confirmed MDR-TB had pyrazinamide resistance ^26^. Ethionamide resistance was found in 13 to 87% of MDR-TB patients depending on the world region ^27^^,^^28^. Other studies in Medellín have reported that 33% of MDR isolates showed cross-resistance between isoniazid and ethionamide suggesting the need to confirm the susceptibility to ETH and PZA before considering their use in the treatment of MDR-TB patients ^29^. Our results showed that 8% of MDR-TB isolates became XDR during treatment or were XDR-TB from the beginning. Among them mortality was high (66%), similar to that reported in other studies, and they were associated with diabetes mellitus, malnutrition, and drug addiction ^30^.

We could not assess a clear relationship between the MDR-TB treatment scheme and its outcome due to the variability of schemes prompted by drug availability, adverse reactions, and initial drug resistance, which is a limitation of this study. Despite a moderate treatment success obtained in this cohort, there was a flaw in the integration of the medical, nutritionist, psychologist, and social work care needed for the comprehensive management of patients. Nevertheless, this program served the purpose of increasing the availability of second-line drugs required for the proper management of MDR-TB patients.

In conclusion, the early recognition of factors that can influence treatment outcomes is essential for the successful treatment of patients with MDR-TB. Knowing these factors would help to focus on their care and should become a high priority for any tuberculosis program. The interdisciplinary care of patients, besides expert medical management and the support of an experienced laboratory, should respond to the nutritional needs of patients, their comorbidities, and the social and psychological factors that affect the final success of the treatment. In the quest for better drugs and treatment schemes for MDR-TB, it is also essential to pay attention to factors beyond the interaction microbe-drug to improve the beneficial impact of new developments in the treatment of these patients and the future of MDR-TB control.
